# Prenatal and early life exposure to air pollution and the risk of severe lower respiratory tract infections during early childhood: the Espoo Cohort Study

**DOI:** 10.1136/oemed-2023-109112

**Published:** 2024-04-10

**Authors:** Abate Bekele Belachew, Aino K Rantala, Maritta S Jaakkola, Timo T Hugg, Mikhail Sofiev, Jaakko Kukkonen, Jouni J K Jaakkola

**Affiliations:** 1 Center for Environmental and Respiratory Health Research, University of Oulu, Oulu, Finland; 2 Biocenter Oulu, University of Oulu, Oulu, Finland; 3 Finnish Meteorological Institute, Helsinki, Finland; 4 Centre for Climate Change Research (C3R), University of Hertfordshire, Hertfordshire, UK

**Keywords:** Air pollution, Epidemiology, Public health

## Abstract

**Background:**

There is inconsistent evidence of the effects of exposure to ambient air pollution on the occurrence of lower respiratory tract infections (LRTIs) in early childhood. We assessed the effects of individual-level prenatal and early life exposure to air pollutants on the risk of LRTIs in early life.

**Methods:**

We studied 2568 members of the population-based Espoo Cohort Study born between 1984 and 1990 and living in 1991 in the City of Espoo, Finland. Exposure assessment was based on dispersion modelling and land-use regression for lifetime residential addresses. The outcome was a LRTI based on data from hospital registers. We applied Poisson regression to estimate the incidence rate ratio (IRR) of LTRIs, contrasting incidence rates in the exposure quartiles to the incidence rates in the first quartile. We used weighted quantile sum (WQS) regression to estimate the joint effect of the studied air pollutants.

**Results:**

The risk of LRTIs during the first 2 years of life was significantly related to exposure to individual and multiple air pollutants, measured with the Multipollutant Index (MPI), including primarily sulphur dioxide (SO_2_), particulate matter with a dry diameter of up to 2.5 µm (PM_2.5_) and nitrogen dioxide (NO_2_) exposures in the first year of life, with an adjusted IRR of 1.72 per unit increase in MPI (95% CI 1.20 to 2.47). LRTIs were not related to prenatal exposure.

**Conclusions:**

We provide evidence that ambient air pollution exposure during the first year of life increases the risk of LRTIs during the first 2 years of life. SO_2_, PM_2.5_ and NO_2_ were found to contribute the highest weights on health effects.

**WHAT IS ALREADY KNOWN ON THIS TOPIC**

- Prenatal and early childhood exposure to ambient air pollution increases the risk of asthma and impairs lung function during childhood and later in life.
- However, there is no consistent evidence on the effects of prenatal and early life exposure to ambient air pollution on the occurrence of lower respiratory tract infections (LRTIs).
- Furthermore, no previous study has investigated the potential effect of a mixture of air pollutants on LRTIs in the first 2 years of life.

**WHAT THIS STUDY ADDS**

- Exposure to ambient air pollution, predominantly including sulphur dioxide, particulate matter with a dry diameter of up to 2.5 µm and nitrogen dioxide, during the first year of life increases the risk of LRTIs in the first 2 years of life.

**HOW THIS STUDY MIGHT AFFECT RESEARCH, PRACTICE OR POLICY**

- Our findings provide evidence that exposure to ambient air pollutants during the first year of life increases the risk of LRTIs during the first 2 years of life.
- This suggests that young children should be better protected against exposure to air pollution.

## Introduction

Lower respiratory tract infections (LRTIs) continue to be the leading cause of morbidity and mortality in younger children worldwide.^1 2^ Air pollution has been reported to increase the risk of respiratory diseases on a global scale.^3^ Pregnant women and young children constitute subgroups most susceptible to the adverse effects of air pollutants.^4 5^

In a systematic literature search, we identified altogether 13 cohort studies that assessed prenatal and/or long-term postnatal exposure to air pollution and the risk of early childhood LRTIs (online supplemental appendix 1 and table S1). In summary, the evidence on the risk of early childhood LRTIs related to prenatal and early life exposure to air pollution is still controversial.

10.1136/oemed-2023-109112.supp1 Supplementary data

Air pollution is a complex mixture of solid particles and gases in the air; it is therefore challenging to assess the independent effects of air pollutants.^6^ Recent developments in the analysis of multipollutant compounds offer new approaches to assess the health effects of complex varieties of air pollution.^7^ Based on our systematic search no previous studies have assessed the mixture effects of multiple air pollutants on the risk of LRTIs. Such models could improve understanding the overall synergistic or antagonistic effects of air pollutants and identifying the most important air pollutants contributing to the causation of LRTIs. We assessed the potential association between the exposures to multiple ambient air pollutants during the prenatal period and first year of life and the risk of LRTIs during the first 2 years of life.

## Methods

### Study design and population

We applied a population-based prospective study, the Espoo Cohort Study (ECS).^8^ The source population consisted of all the children who were born between 1 January 1984 and 31 March 1990, and were living in 1991 in the city of Espoo, Finland. Espoo is a municipality with a total population of 279 000 located on the western border of Helsinki, the capital of Finland. A random sample of children living in Espoo in 1991 was taken from the roster of Statistics Finland. A total of 2568 children (response rate 80.3%) whose parents filled in the ECS questionnaire formed the baseline study population.

### Exposure assessment

The individual-level exposure to ambient air pollutants during the prenatal period and first year of life was measured based on the concentrations at all the residential addresses from conception to birth and from birth to the first year of life while accounting for residential mobility. We modelled hourly values of air pollutants for a computational spatiotemporal grid for a time period from the conception to the end of the follow-up period of the Espoo cohort. Exposure for each member of the cohort was calculated individually using the location and the time spent in all of their residences.

We obtained the data on air pollutants of interest from the Finnish Meteorological Institute (FMI). The pollutant data were based on a cascade of nested simulations of the System for Integrated modeLling of Atmospheric coMposition (SILAM).^9^ To evaluate the global and regional background levels of air pollutants, the model was first executed on a global scale and then zoomed to Europe and Northern Europe, reaching the spatial resolution of 0.1° × 0.1°.

We retrieved the home coordinates from the Population Register Centre of Finland. The Geographical Information System was used to extract daily levels of air pollutants at these home coordinates. The exposures of interest were atmospheric air pollutants during pregnancy and the first year of life. The air pollutants of interest constituted of the fine particulate matter with a dry diameter of up to 2.5 µm (PM_2.5_), the particulate matter with a dry diameter of up to 10 μm (PM_10_), sulphur dioxide (SO_2_), nitrogen dioxide (NO_2_), carbon monoxide (CO) and ozone (O_3_).

### Health outcomes

The health outcome of interest was a LRTI requiring hospitalisation. In this study, LRTIs included clinician-diagnosed pneumonia and acute bronchitis. We used the National Hospital Discharge Register to extract information on all LRTIs that had led to hospitalisation. The National Hospital Discharge Register included the dates and causes of all hospital admissions requiring an overnight stay that had occurred since January 1969. We applied the International Classification of Diseases (ICD) eighth edition codes for the diagnoses until 1986 and ICD ninth edition codes for the diagnoses between 1987 and 1995 (online supplemental table S2). The personal identification number was used to link the register-based data with the cohort data. Incidence rates for the LRTIs were calculated using the number of infections during the first 2 years of life as the numerator and the time at risk in person-years as the denominator.

### Covariates

We adjusted for covariates that are reported to be potential determinants of LRTIs in scientific literature. These included secondhand tobacco smoke,^10^ maternal smoking during pregnancy,^11^ family socioeconomic status (based on the highest parental education and occupation),^12^ sex of the child, preterm birth, duration of breast feeding^11 13^ and indoor mould exposure.^8^ The information on these covariates was obtained from the baseline assessment that was conducted at the age of 1–6 years.

### Statistical analysis

We applied Poisson regression analysis to estimate the incidence rate ratio (IRR) as the measure of the effect of air pollutants on the risk of LRTIs. Incidence rates of LRTIs in the highest quartiles (second, third and fourth) of air pollutant exposure during pregnancy and the first year of life were compared with the reference, that is, the lowest quartile. We fitted single, two and three pollutant models. We tested potential presence of linear trends in the risk of LRTIs with increasing air pollution levels. To support causal inference, we took into account the temporal relation between exposure to air pollution and the occurrence of LRTIs. We modelled at individual level the association of air pollution levels preceding or occurring concurrently with the episodes of LRTIs.

We conducted a sensitivity analysis to ensure the robustness of the study results. We conducted an analysis using only term-born study children. Furthermore, to take into account the observed strong collinearity between pollutants (online supplemental table S3), we used weighted quantile sum (WQS) regression that can reduce the impact of collinearity and helps in estimating the cumulative effect of pollutants.^14^ The individual weights indicate the relative importance (in %) of each exposure in the pollutant mixture-outcome association. The sum of the included pollutant weights is 100%. WQS implementation is presented in the supplementary file (online supplemental appendix 2). All analyses were conducted using Stata V.16 (StataCorp, College Station, Texas, USA) and R V.4.2.3.

## Results

### Characteristics of the study population

The characteristics of the study population are presented in table 1. A comparable number of boys and girls participated in the ECS, 48.9% and 51.1%, respectively. More than half (53.7%) of the children were breast fed for eight or more months and 7.8% were born preterm (ie, <37 weeks). About 86.0% of mothers had no smoking history during pregnancy and 26.1% of children were from a family with low socioeconomic status.

**Table 1:** Characteristics of the study population, the Espoo Cohort Study (n=2568)*

Characteristic	n	%
Sex of child		
Female	1257	48.9
Male	1311	51.1
Duration of breast feeding, months		
<4	481	19.3
4–7	670	26.9
≥8	1337	53.7
Duration of pregnancy, weeks		
<37	195	7.8
≥37	2301	92.2
Maternal smoking during pregnancy		
No	2199	85.8
Yes	364	14.2
Family socioeconomic status		
Low	667	26.1
Medium/high	1889	73.9
Atopy		
No	2276	88.6
Yes	292	11.4
Parental atopy		
No	1697	66.1
Yes	871	33.9
Secondhand tobacco smoke exposure		
No	2171	84.5
Yes	397	15.5
Indoor mould exposure		
No	2428	94.6
Yes	138	5.4

*Missing value in the duration of breast feeding (n=79), duration of pregnancy (n=72), maternal smoking (n=5), family socioeconomic status (n=12) and indoor mould exposure (n=2).

### Exposure distributions

There were no major differences in the average air pollutant exposures during pregnancy or the first year of life (table 2). The average concentrations of air pollutants in each quartile of exposure distribution were presented in online supplemental table S4.

**Table 2:** Concentrations (µg/m^3^) of air pollutants during the two exposure periods, Espoo, Finland

Pollutants	Mean±SD	Minimum	25th percentile	50th percentile	75th percentile	IQR	Maximum
During entire pregnancy (n=2525)							
PM_2.5_	19.62±4.50	3.01	16.93	19.53	22.10	5.17	38.48
PM_10_	21.35±5.12	3.17	18.47	21.15	24.06	5.59	43.78
NO_2_	8.72±2.49	0.43	7.34	8.93	10.26	2.92	17.68
CO	363.80±63.49	147.81	325.50	364.90	406.52	81.02	594.80
SO_2_	11.10±5.49	0.53	7.77	10.56	13.27	5.5	44.76
O_3_	50.27±5.91	23.68	45.74	50.57	54.95	9.21	78.06
First year of life (n=2532)							
PM_2.5_	19.21±3.82	3.09	17.06	19.07	21.31	4.25	37.88
PM_10_	20.95±4.35	3.24	18.63	20.70	23.18	4.55	43.85
NO_2_	8.66±2.20	0.48	7.36	8.82	10.05	2.69	17.02
CO	362.37±55.01	159.97	325.88	371.02	397.92	72.04	574.98
SO_2_	10.43±4.76	0.58	7.57	9.85	12.59	5.02	43.16
O_3_	51.05±2.85	35.08	49.14	50.97	53.16	2.19	81.04

CO, carbon monoxide; NO_2_, nitrogen dioxide; O_3_, ozone; PM_10_, particulate matter with a diameter of 10 μm; PM_2.5_, particulate matter with a diameter of 2.5 μm; SO_2_, sulfur dioxide.

A positive and strong correlation was found between the exposure to air pollutants during the studied exposure periods, except for O_3,_ for which there was a negative correlation with the other pollutants (online supplemental table S3).

### Incidence of LRTIs

The incidence rate of LRTIs requiring hospitalisation was 1.47 per 100 person-years in girls and 1.98 per 100 person-years in boys. Thus, the incidence rate was slightly higher for boys than for girls, but the difference was not statistically significant (online supplemental table S5).

Table 3 presents the incidence rate of LRTIs according to the quartiles of air pollution exposures. The incidence rate of LRTIs was found to increase with increasing levels of air pollution exposures during the first year of life. However, no such relation was detected with prenatal air pollution exposures.

**Table 3:** Incidence rate (IR) of hospital-diagnosed lower respiratory tract infections during the first 2 years of life by quartiles (Q) of the mean air pollutant exposures during pregnancy and in the first year of life, Espoo Cohort Study

Exposures	n	Prenatal exposure	First-year exposure	
		IR (95% CI)	n	IR (95% CI)
PM_2.5_				
Q1	17	1.36 (0.79 to 2.18)	14	1.11 (0.60 to 1.86)
Q2	29	2.29 (1.53 to 3.28)	18	1.42 (0.84 to 2.25)
Q3	20	1.57 (0.96 to 2.42)	24	1.90 (1.21 to 2.82)
Q4	22	1.74 (1.09 to 2.64)	32	2.53 (1.73 to 3.57)
PM_10_				
Q1	20	1.57 (0.96 to 2.42)	15	1.18 (0.66 to 1.95)
Q2	26	2.04 (1.33 to 2.99)	17	1.34 (0.78 to 2.15)
Q3	21	1.68 (1.04 to 2.56)	25	1.97 (1.28 to 2.92)
Q4	21	1.68 (1.04 to 2.57)	31	2.45 (1.66 to 3.48)
NO_2_				
Q1	20	1.61 (0.98 to 2.48)	19	1.50 (0.90 to 2.34)
Q2	29	2.32 (1.55 to 3.33)	14	1.11 (0.60 to 1.86)
Q3	15	1.15 (0.64 to 1.89)	24	1.90 (1.21 to 2.82)
Q4	24	1.93 (1.24 to 2.87)	31	2.45 (1.66 to 3.48)
CO				
Q1	22	1.74 (1.09 to 2.64)	19	1.50 (0.90 to 2.34)
Q2	22	1.74 (1.09 to 2.64)	17	1.34 (0.78 to 2.15)
Q3	20	1.59 (0.97 to 2.46)	24	1.90 (1.21 to 2.82)
Q4	24	1.89 (1.21 to 2.82)	28	2.21 (1.47 to 3.20)
SO_2_				
Q1	13	1.00 (0.53 to 1.71)	16	1.26 (0.72 to 2.05)
Q2	33	2.65 (1.82 to 3.72)	16	1.27 (0.72 to 2.06)
Q3	21	1.67 (1.03 to 2.56)	19	1.50 (0.90 to 2.34)
Q4	21	1.68 (1.04 to 2.57)	37	2.92 (2.06 to 4.03)
O_3_				
Q1	30	2.39 (1.61 to 3.41)	31	2.45 (1.66 to 3.48)
Q2	21	1.64 (1.02 to 2.51)	26	2.05 (1.34 to 3.01)
Q3	12	0.95 (0.49 to 1.65)	15	1.18 (0.66 to 1.95)
Q4	25	2.01 (1.30 to 2.96)	16	1.26 (0.72 to 2.05)

CO, carbon monoxide; IR, Incidence rate per 100 person-years; n, episodes of infections; NO_2_, nitrogen dioxide; O_3_, ozone; PM_10_, particulate matter with a diameter of 10 μm; PM_2.5_, particulate matter with a diameter of 2.5 μm; SO_2_, sulfur dioxide.

### Air pollution exposure and the risk of LRTIs

After adjusting for potential confounders, none of the prenatal exposure to air pollutants was associated with hospital admissions due to LRTIs in the first 2 years of life (online supplemental table S6). However, in the single pollutant model the incidence rates of LRTI in the highest quartiles of postnatal (the first year) exposures to PM_2.5_, NO_2_ and CO concentrations were higher compared with the lowest quartile, with IRR of 2.92 (95% CI 1.44 to 5.92), 2.83 (95% CI 1.40 to 5.71) and 2.28 (95% CI 1.09 to 4.74), respectively (table 4). Similarly, the occurrence of LRTIs was also related with the first-year exposure to PM_10_ and SO_2_ concentrations, with IRR of 2.82 (95% CI 1.40 to 5.70) and 2.81 (95% CI 1.35 to 5.83), respectively. These findings were consistent when we analysed exposure during postnatal period and episodes of LRTIs at 1–2 years of life (online supplemental table S7).

**Table 4:** Association between exposure to air pollution during the first year of life and incidence rate of lower respiratory tract infections during the first 2 years of life, incidence rate ratio (IRR) as the measure of effect

Exposure	Effect estimates, IRR (95% CI), relative to the first quartile			Per 10 µg/increase
	Quartile 2	Quartile 3	Quartile 4	IRR (95% CI)
PM_2.5_*	1.23 (0.59 to 2.55)	1.86 (0.91 to 3.80)	**2.50 (1.27 to 4.93)**	1.24 (0.63 to 2.43)
PM_2.5_†	1.39 (0.67 to 2.92)	1.66 (0.77 to 3.60)	**2.92 (1.44 to 5.92)**	1.70 (0.84 to 3.43)
+SO_2_	1.77 (0.68 to 4.59)	1.69 (0.50 to 5.67)	1.58 (0.41 to 6.16)	1.23 (0.27 to 5.51)
+O_3_	1.49 (0.64 to 3.47)	1.60 (0.57 to 4.46)	2.56 (0.87 to 7.51)	0.83 (0.32 to 2.19)
+SO_2_ +O_3_	1.90 (0.66 to 5.45)	1.67 (0.42 to 6.56)	1.54 (0.33 to 7.17)	0.85 (0.20 to 3.67)
PM_10_*	1.12 (0.54 to 2.33)	1.90 (0.95 to 3.81)	**2.42 (1.23 to 4.76)**	1.20 (0.65 to 2.19)
PM_10_†	1.23 (0.59 to 2.59)	1.66 (0.79 to 3.51)	**2.82 (1.40 to 5.70)**	1.61 (0.85 to 3.04)
+ CO	1.67 (0.69 to 4.06)	2.26 (0.79 to 6.45)	**3.38 (1.09 to 10.54)**	1.17 (0.37 to 3.70)
+ NO_2_	1.91 (0.75 to 4.85)	2.49 (0.79 to 7.87)	2.97 (0.81 to 10.93)	1.09 (0.25 to 4.74)
+ CO + O_3_	1.63 (0.59 to 4.53)	1.96 (0.53 to 7.27)	2.62 (0.60 to 11.44)	0.68 (0.19 to 2.38)
+ NO_2_ + O_3_	1.83 (0.63 to 5.34)	2.17 (0.54 to 8.67)	2.30 (0.46 to 11.43)	0.62 (0.13 to 2.98)
NO_2_*	0.77 (0.38 to 1.58)	1.85 (0.92 to 3.73)	**2.31 (1.18 to 4.53)**	1.58 (0.41 to 6.17)
NO_2_†	0.83 (0.40 to 1.73)	1.71 (0.81 to 3.61)	**2.83 (1.40 to 5.71)**	2.75 (0.64 to 11.82)
+ PM_10_	0.53 (0.21 to 1.34)	0.89 (0.28 to 2.84)	1.37 (0.35 to 5.32)	3.91 (0.12 to 12.84)
+ O_3_	0.83 (0.38 to 1.82)	1.46 (0.60 to 3.54)	2.28 (0.86 to 6.06)	0.78 (0.11 to 5.56)
+ PM10+ O_3_	0.57 (0.22 to 1.51)	0.96 (0.29 to 3.15)	1.53 (0.37 to 6.37)	2.61 (0.07 to 91.97)
CO *	0.97 (0.49 to 1.92)	1.51 (0.74 to 3.05)	1.79 (0.89 to 3.62)	1.01 (0.96 to 1.06)
CO †	0.92 (0.44 to 1.90)	1.71 (0.82 to 3.58)	**2.28 (1.09 to 4.74)**	1.04 (0.98 to 1.10)
+ PM_10_	0.59 (0.24 to 1.45)	0.87 (0.31 to 2.43)	0.87 (0.27 to 2.84)	1.05 (0.94 to 1.16)
+ O_3_	0.84 (0.39 to 1.79)	1.33 (0.58 to 3.05)	1.51 (0.61 to 3.71)	1.00 (0.93 to 1.07)
+ SO_2_	0.53 (0.19 to 1.45)	0.61 (0.19 to 1.98)	0.39 (0.10 to 1.49)	1.03 (0.94 to 1.13)
+ SO_2_ + O_3_	0.58 (0.21 to 1.59)	0.68 (0.20 to 2.29)	0.43 (0.11 to 1.69)	1.02 (0.93 to 1.12)
+ PM_10_+ O_3_	0.64 (0.25 to 1.59)	0.94 (0.32 to 2.70)	0.95 (0.28 to 3.22)	1.04 (0.93 to 1.15)
SO_2_*	0.75 (0.36 to 1.57)	1.21 (0.57 to 2.59)	**2.49 (1.24 to 5.02)**	1.40 (0.73 to 2.69)
SO_2_†	0.80 (0.38 to 1.69)	1.16 (0.52 to 2.61)	**2.81 (1.35 to 5.83)**	1.91 (0.92 to 3.96)
+ CO	1.28 (0.47 to 3.47)	2.05 (0.60 to 6.99)	6.37 (1.68 to 24.15)	1.87 (0.65 to 5.39)
+ NO_2_	1.52 (0.55 to 4.19)	2.68 (0.72 to 10.00)	7.89 (1.74 to 35.72)	2.05 (0.63 to 6.66)
+ O_3_	0.81 (0.37 to 1.77)	1.14 (0.44 to 2.95)	2.71 (0.98 to 7.51)	0.99 (0.35 to 2.79)
+ CO + O_3_	1.16 (0.40 to 3.37)	1.79 (0.44 to 7.32)	5.62 (1.14 to 27.80)	1.02 (0.27 to 3.86)
+NO_2_ + O_3_	1.40 (0.48 to 4.09)	2.37 (0.55 to 10.12)	6.71 (1.21 to 37.05)	1.14 (0.28 to 4.71)
O_3_*	0.81 (0.48 to 1.38)	0.49 (0.26 to 0.93)	0.49 (0.26 to 0.92)	0.49 (0.22 to 1.11)
O_3_†	0.79 (0.45 to 1.39)	0.54 (0.27 to 1.07)	0.55 (0.29 to 1.06)	0.58 (0.24 to 1.36)
+ SO_2_	1.11 (0.58 to 2.12)	0.82 (0.35 to 1.95)	0.85 (0.33 to 2.19)	0.25 (0.07 to 0.91)
+ NO_2_	0.94 (0.49 to 1.81)	0.65 (0.29 to 1.47)	0.62 (0.26 to 1.50)	0.29 (0.09 to 0.94)
+ CO	0.83 (0.44 to 1.54)	0.58 (0.27 to 1.24)	0.56 (0.25 to 1.29)	0.33 (0.10 to 1.02)
+ PM_2.5_	1.02 (0.53 to 1.94)	0.76 (0.31 to 1.83)	0.87 (0.32 to 2.36)	0.26 (0.08 to 0.86)
+ SO_2_ + CO	1.08 (0.56 to 2.09)	0.89 (0.38 to 2.08)	0.91 (0.35 to 2.36)	0.25 (0.07 to 0.91)
+ PM_10_ + NO_2_	0.98 (0.50 to 1.93)	0.75 (0.31 to 1.80)	0.82 (0.30 to 2.25)	0.25 (0.08 to 0.85)
+ PM_2.5_ + SO_2_	1.14 (0.58 to 2.24)	0.83 (0.33 to 2.07)	0.94 (0.33 to 2.63)	0.23 (0.06 to 0.84)
+ NO_2_ + SO_2_	1.03 (0.53 to 2.01)	0.87 (0.37 to 2.05)	0.87 (0.33 to 2.26)	0.25 (0.07 to 0.90)

*Single pollutant models, adjusted only for prenatal exposure of respective pollutant.

†Adjusted for prenatal exposure of respective pollutants and covariates in table 1.

CO, carbon monoxide; IRR, incidence rate ratio; NO_2_, nitrogen dioxide; O_3_, ozone; PM_10_, particulate matter with a diameter of 10 μm; PM_2.5_, particulate matter with a diameter of 2.5 μm; SO_2_, sulfur dioxide.

**Figure 1:**
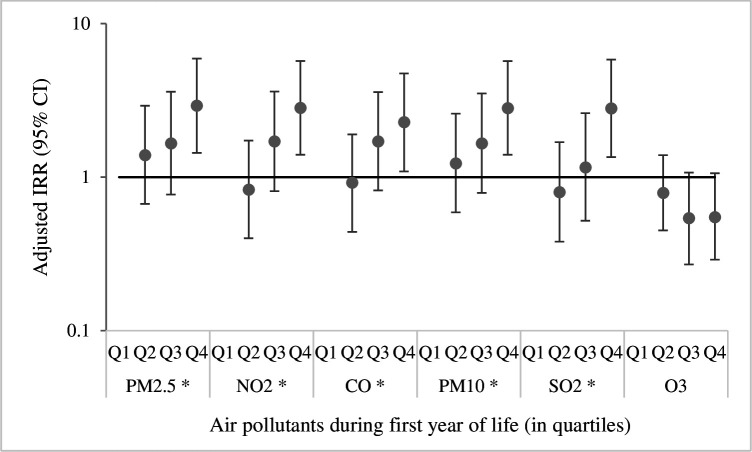
Adjusted incidence rate ratio (IRR) of early childhood respiratory infection in higher quartiles of air pollution exposures compared with the lowest quartile (horizontal reference line). *Indicates significant trend in effects (p-trend<0.05). CO, carbon monoxide; NO_2_, nitrogen dioxide; O_3_, ozone; PM_10_, particulate matter with a diameter of 10 µm; PM_2.5_, particulate matter with a diameter of 2.5 µm; Q_1 – 4_, first to fourth quartiles; SO_2_, sulphur dioxide.

The multipollutant models were fitted by including one traffic-related (PM_2.5_, NO_2_, CO) and one stationary fossil fuel combustion-related pollutant (PM_10_, SO_2_) and/or O_3._ However, the effect estimates in the multipollutant models were systematically lower than in the single-pollutant models and they appeared more imprecise, which is explained by a high degree of correlation between the pollutants, as presented in online supplemental table S3. There were significant linear trends in the effect of air pollutant exposure quartiles for all air pollutant exposures and LRTIs, except for O_3_ exposure (figure 1).

In WQS analysis, the incidence rate of LRTIs increased by 72% (IRR=1.72, 95% CI 1.20 to 2.47) for a unit increase in the multipollutant index (MPI) in the first year of life, which represents quartiles of multiple air pollutants, each weighted according to its contribution to the overall association with the outcome. The weights for different air pollutants contributing to the pollutant mixture index were SO_2_ (contribution 36.3%), PM_2.5_ (30.8%), NO_2_ (12.8%), O_3_ (8.6%), PM_10_ (7.5%) and CO (4.0%). Prenatal air pollutant exposures did not show similar effects on LTRIs. The MPI for the exposures during the entire prenatal period was not statistically significant (IRR=0.68, 95% CI 0.45 to 1.05). This finding was consistent with observed non-significant exposure-outcome associations in the single and multipollutant models (online supplemental table S6).

## Discussion

### Main results

We assessed the effects of air pollution exposure on the risk of LRTIs by estimating the associations between single and multiple air pollutant exposures during prenatal and infancy periods and the incidence rate of LRTIs during the first 2 years of life in the population-based ECS. We applied a novel approach to estimate the effect of a complex mixture of correlating pollutants using the MPI. The risk of LRTIs during the first 2 years of life was linearly related to the MPI, the major contributions being from SO_2_, PM_2.5_ and NO_2_ exposures during the first year of life. There was no consistent evidence of any effect of prenatal air pollutant exposure on the risk of LRTIs.

### Validity of results

This study was based on a population-based cohort study consisting of a random sample of children from the Roster of Statistics Finland, with a high response rate (80.3%) at baseline. Hence, the risk of potential selection bias was relatively small.

Exposure to air pollution was assessed at the individual level, independently from the assessment of the health outcome occurrences. This should minimise the likelihood of information bias. We took into account the potential changes in the residential addresses during the entire pregnancy and first year of life, hence potential exposure misclassification and related information bias due to mobility is unlikely. The exposure data were estimated with high resolution—the temporal resolution of 1 hour and the spatial resolution of approximately 10 km of SILAM simulations downscaled to hundreds of metres. These high-resolution estimations considered both the emissions from vehicular traffic and small-scale combustion sources by using the road network dispersion model (Contaminants in the Air from a Road, CAR-FMI), and the multiple-source Gaussian dispersion model (Dispersion Model for Stationary Point, Area and Volume Sources, UDM-FMI).^15^ The spatial resolution of exposure data was relatively coarse. In the study area, the spatial resolution applied in this study corresponded to around 5.5 km in the east-west direction, and 11.1 km in the north-south direction. It was not possible to use urban scale models for such a long period of time, including numerous pollutants. Kukkonen *et al* 15 have presented a multidecadal (from 1980 to 2014) assessment on an urban scale for the Helsinki Metropolitan Area; however, that study addressed only the concentrations of PM_2.5_. In that study, the distances between the receptor points varied from 25 m near roads to 200 m in rural areas.

The outcome of interest, an episode of LRTI requiring hospitalisation including acute bronchitis and pneumonia, was defined based on ICD codes from clinical diagnoses, which reduced the likelihood of outcome misclassification. The ICD codes given by health professionals were considered to provide a more objective measure compared with parental reports. The latter may be influenced by recall bias, potentially leading to outcome misclassification.

We conducted several sensitivity analyses, including the use of gestational age (ie, analysing separately term and preterm infants) and the sex of the newborn. The results were comparable in both cases (online supplemental table S8 and S9). In our previous report on the ECS, we showed that asthma is related to an increased risk of getting respiratory tract infections in both early childhood and young adulthood.^16^ Thus, part of the effect of air pollution exposure on the risk of LRTIs could be mediated by emerging or existing asthma. We could not elaborate the role of asthma in the causal path, because most of the asthma diagnoses were made after the age of 2 years.

We were also able to control for several potential confounders, including secondhand tobacco smoke, maternal smoking during pregnancy, family socioeconomic status (based on the highest parental education and occupation), sex of the child, preterm birth, duration of breast feeding, and indoor mould exposure, that have been reported to increase the risk of LRTIs. Information on potential confounders was from the data collection in 1991 when the children were from 1 year to 7 years old. Information on some of these potential confounders was expected to be accurate (eg, family socioeconomic status), but for others (eg, indoor mould exposure) time distance may have influenced the accuracy of the information, but the influence was expected to be non-differential with respect to the studied relations. In addition, we did not have information on some potential confounders, such as type of furniture or recent refurbishment, which have recently been found to be determinants of LRTIs.^17^

According to a systematic literature search, the present study is the first to consider the effect of the exposure to multiple simultaneous air pollutants on the risk of early childhood LRTIs. Previous studies have used mainly single-pollutant models.^18–24^ We identified two studies that used multipollutant models to estimate each pollutant’s independent effects on the risk of LRTIs.^25 26^ The results obtained using single pollutant models are likely to be influenced by residual confounding, as those do not take into account potential confounding and synergism between the effects of multiple pollutants. On the other hand, studies using multipollutant models might suffer from multicollinearity among the pollutants, which could also lead to biased effect estimates. Moreover, as urban air pollution is always a complex mixture of particles and various gases, an analysis based on a multipollutant mixture approach is likely to provide the most valid effect estimate on the association between air pollution and LRTIs. By using WQS regression analysis, we were able to estimate the effect of the ambient air pollution mixture on the risk of early childhood LRTIs. WQS regression combines correlated pollutants into a single index, effectively controlling the multicollinearity effect. This unification ensures that the estimates produced are reliable.

### Synthesis with previous knowledge

Prenatal and early childhood periods have been identified as critical periods for the effects of environmental exposures on adverse health outcomes later in life, including respiratory infections.^27^ There is substantial evidence showing an association between prenatal and early life exposure to air pollution and adverse respiratory health later in life,^28 29^ but little information on effects on early childhood respiratory infections. In a recent systematic review, Gheissari *et al* 5 reported a significant association between early life exposure to air pollution and adverse respiratory outcomes.^5^ Among the studies that were included in that review, only a few had assessed the risk of LRTIs as the outcome, while some of these showed inconsistent results.^23^ Almost all these studies assessed only the effects of short-term exposure to air pollution in early life. A meta-analysis based on 17 studies conducted until January 2017 reported a significant association between short-term exposure to ambient air pollution and increased hospitalisation due to pneumonia in children who were younger than 18 years of age.^30^ None of these studies investigated potential health effects related to exposure to the air pollutant mixture. In a recent systematic review, which included altogether eight case-control and case-cross-over studies, the effect of short-term and long-term exposure to ambient air pollution on the risk of infant bronchiolitis was inconclusive.^31^ In addition, previous cohort studies reported inconsistent findings about the effect of prenatal and early life exposure to air pollution on the risk LRTIs during early childhood (online supplemental table S1).

In the present study, the first-year exposure to all studied air pollutants, except for O_3_, showed a linear relationship with an increasing incidence rate of LRTIs from the lowest quartile (the reference) to the highest quartile of exposure concentrations of air pollutants. The analyses showed that exposure to SO_2_, PM_2.5_ and NO_2_ contributed most to the association between air pollutant mixture and increased incidence rate of LRTIs. The result of the present study on the effect of particulate matter exposure during early life (ie, in infancy) and increased rate of LRTIs is consistent with some previous studies.^21 32 33^ Mehta *et al* 32 reported a significant relation (effect estimate: 1.12, 95% CI 1.03 to 1.30) between long-term exposure to PM_2.5_ and LRTIs in the first 2 years of life.^32^ A cohort study from the USA reported that first-year of life exposure to PM_2.5_, NO_x_ and CO increased the risk of bronchiolitis in children younger than 2 years of age.^21^ Our findings are also consistent with a study conducted in slightly older children, that is, preschool children from China, who were exposed to substantially higher air pollution levels compared with our study. They showed that an IQR increase in NO_2_ exposure during the first year of life was related to a significantly increased odds of pneumonia, adjusted OR being 1.56 (95% CI 1.14 to 2.20).^25^

The present study did not indicate any association between prenatal exposure to ambient air pollution and LRTIs during the first 2 years of life when adjusting for early life exposure. Some previous cohort studies from Israel and Poland showed evidence of a significant relationship between exposure to PM_2.5_ during pregnancy and early childhood respiratory infections.^18 24^ The difference in comparison to our results could be explained at least partly by higher air pollution levels in the Israeli^18^ and Polish^24^ studies with the average PM_2.5_ during pregnancy being 22 μg/m^3^ and 43.37 μg/m^3^, respectively. Similarly, a case-control study from China showed a positive, significant association between prenatal exposure to SO_2_ and pneumonia during 0–14 years of age.^34^ In contrast, the evidence on prenatal exposure to NO_2_ and the risk of LRTIs has been weak.^33 35^ Further longitudinal studies are still important to strengthen the hypothesis that prenatal air pollution exposure increases the risk of early childhood respiratory infections.

The present study is based on air pollution levels from 1983 to 1991 in the Helsinki Metropolitan area. Kukkonen *et al* 15 reported that the highest concentrations of PM_2.5_ in this region occurred in the 1980s; these have since decreased to about to a half of the highest values. They also reported that local vehicular emissions of fine particulate matter were about five times higher in the 1980s, compared with the emissions during the early 2010s. The local small-scale combustion emissions of PM_2.5_ have increased slightly over time since the 1980s. In conclusion, this study corresponds to a period of substantially higher atmospheric pollutant concentrations, compared with the present-day situation in this area. On the other hand, comparable or even higher levels of air pollution are still nowadays commonly occurring in, for example, numerous European major cities. The results of this study are therefore fairly well generalisable for a large fraction of European major cities, compared with studies conducted in modern-day Nordic cities, in which the pollutant levels are moderate in a European comparison. Recent results of the particulate concentrations in three Nordic capitals have been presented by Kukkonen *et al*.^36^

### Biological plausibility

Prenatal and early life exposure to air pollution may increase susceptibility to respiratory infections through various pathways.^37^ Respiratory system encounters first any air pollution exposure. Fine particulate matter and gaseous air pollutants can infiltrate and deposit even within the smaller airways; such an exposure may hamper the immune system.^38^ Such air pollutants may move beyond the respiratory system through the air-blood barrier and reach the blood circulation through the placenta. Air pollutant exposures can induce oxidative stress and impair the developing immune system.^37 39^ Furthermore, air pollutants may induce epigenetic changes which can lead to increased susceptibility to respiratory infections in childhood.^37^

## Conclusions

The present study provides novel evidence that exposure to a mixture of ambient air pollutants during the first year of life increases the risk of LRTIs during the first 2 years of life. An MPI, which was based predominantly on SO_2_, PM_2.5_ and NO_2_ exposures, described exposure to multiple pollutants simultaneously and was significantly related to an increased risk of LRTIs. The urban ambient air pollutants are interrelated, and always occur as a complex mixture; we therefore recommend such a mixture effect analysis for an assessment of their joint effects on health. The results suggest that it is important to include the concentrations of all the main air pollutants in setting air pollution regulations, in comparison to the traditional single-pollutant approach.

## Data Availability

Data are available upon reasonable request. The data sets generated and/or analysed during the current study are not publicly available due to issues of confidentiality, but are available from the corresponding author on reasonable request.
