# Supplementary material for: Prenatal and early life exposure to air pollution and the risk of severe lower respiratory tract infections during early childhood: the Espoo Cohort Study
Source: Occup Environ Med. 2024 Apr 10;81(4):209–16. doi: 10.1136/oemed-2023-109112 (PMC11103339; doi:10.1136/oemed-2023-109112)
Supplement: Supplementary data [file oemed-2023-109112supp001.pdf]

Supplemental Material

Prenatal and early-life exposure to air pollution and risk of severe lower respiratory tract infections during early childhood: the Espoo Cohort Study

Appendix 1: A systematic review of literature on the effects of perinatal exposure to air pollution and the risk of LRTIs during early childhood.

We conducted a systematic literature search on PubMed from inception to March 12, 2023, aiming to identify previous epidemiologic studies that assessed early-life air pollution exposures and the risk of respiratory tract infections during early childhood. We developed a query with keywords and search terms related to air pollution, early childhood, and respiratory tract infections. The query was made using Boolean logic (AND/OR/NOT) as ((air pollution) OR (air pollutants) OR (traffic-related pollution)) AND ((intrauterine) OR (pregnant) OR (maternal-fetal exchange) OR (prenatal) OR (antenatal) OR (maternal exposure) OR (early childhood) OR (infant) OR (postnatal)) AND ((respiratory tract infections) OR (lower respiratory tract infection) OR (pneumonia) OR (bronchitis) OR (bronchiolitis)) NOT ((asthma[Mesh])) NOT ((COVID-19[Mesh])) AND (humans[Filter]). The search identified, altogether, 740 journal articles. The inclusion criteria were the following: i) an original study of any epidemiologic design; ii) estimation of the risk of LRTIs; iii) in early childhood; iv) in relation to ambient air pollution exposure; v) during the perinatal period (pregnancy and first year of life). Based on the inclusion criteria, we excluded 674 irrelevant articles during the title and abstract screening. We found 67 studies that were eligible for a full-text review. More than half of these studies (n = 36) assessed only the effects of short-term exposure to ambient air pollution. Further 10 studies were excluded due to the following reasons: i) did not provide estimates for exposure-outcome association (n = 5); ii) used respiratory symptoms only as the outcome (n = 3), iii) used distance to traffic or traffic density as the exposure (n = 1), and iv) did not use an eligible study design (n = 1). One article was found after reviewing the references of the eligible studies. We updated our search on October 9, 2023, and we found two more articles. Finally, 24 original studies that assessed prenatal and/or first-year exposure to ambient air pollution and the risk of early childhood respiratory tract infections were identified. However, only 13 of them were cohort studies. The number of studies that assessed the effects of prenatal, postnatal, and both exposures on RTIs were five, three, and five, respectively.

Table S1: Previous cohort studies that assessed the effect of prenatal and first-year exposure to ambient air pollution on the risk of lower respiratory tract infections.

First Author, Year	Location, climatic zone [a]	Study population, age	Method of exposure assessment	Studied exposures	Exposure time periods	Outcome	Effect estimate, 95% CI	Remarks: Modifications
**Lu 2023[1]**	Changsha, China; Cfa	Children, 3-6 years	Individual-level exposure, using inverse distance weighting	$PM_{2.5}$, PM_{10}, SO_2, NO_2, and CO	Prenatal and Postnatal (first year)	Pneumonia, parental report	Prenatal:<br>$PM_{2.5}$ – 1.17, 1.04, 1.30<br>PM_{10} – 1.07, 1.00, 1.14<br>NO_2 – 1.10, 1.00, 1.22<br><br>Postnatal:<br>PM10 – 1.11, 1.03, 1.21	effect estimates are per interquartile range (IQR) increment of exposure
**Goshen 2020[2]**	Beer-Sheva, Southern Israel; BSh	n= 57,331, **infants** (Arab-Bedouin, Jewish ethnicity)	Individual-level exposure	$PM_{2.5}$: quartiles<br>Mean(range): Jewish<br>22.3(13.0–60.4) μg/m^3<br>Q_4 – 23.7–60.4 μg/m^3<br>Arab-Bedouin<br>22.0(13.5–77.0) μg/m^3<br>Q_4 – 23.1–77.0 μg/m^3	Prenatal	First time hospitalization for bronchitis or pneumonia, ICD-9	Arab-Bedouin<br>1st trimester<br>Q_4 – **1.31, 1.08–1.60**<br>2nd trimester<br>Q_4 – **1.34, 1.09–1.66**	The association was not statistically significant for the **Jewish** population
**Yang 2020[3]**	Seoul, South Korea; Dfa	n = 930, from **birth to 3** years	Individual-level exposure, IDW	$PM_{2.5}$, Highest tertile	Prenatal	Doctor-diagnosed LRTIs, parental report	at 2 years: Middle vs low tertile – 0.98, 0.91, 1.05<br>High vs low tertile – 0.98, 0.91-1.06	Similar result at 1st and third years. But 3rd trimester exposure showed significant effect
**Liu 2020[4]**	Shanghai, China; Cfa	n = 3,177, **4–6** years	Residence-district period average concentrations	IQR increase<br>Mean (Range):<br>Prenatal<br>SO2 - 45.7 (22.6–71.1) μg/m^3<br>NO_2 – 56.0 (33.6 – 74.1) μg/m^3<br>PM_{10} – 83.1 (51.9 – 118.9) μg/m^3<br>Postnatal<br>SO_2 – 44.9 (27.2 – 57.9) μg/m^3.<br>NO_2 – 55.4 (36.0–67.1) μg/m^3.<br>PM_{10} - 82.9 (69.2–96.6) μg/m^3.	Prenatal<br><br>Postnatal (infancy)	Ever had pneumonia, parent reported	Prenatal:<br>SO_2 – 1.04, 0.80-1.34<br>NO_2 - 1.24, 0.90-1.68<br>PM_{10} – 1.04, 0.84–1.31<br>Postnatal:<br>SO_2 – 1.00, 0.79–1.27<br>NO_2 – **1.56, 1.14–2.20**<br>PM_{10} – 0.99, 0.73–1.37	All estimates were from multipollutant exposure.
**Gutiérrez 2018[5]**	Valencia, Spain; BSk	n =624, **2 years**	Individual-level exposure	NO_2, per 10 μg/m^3 increase<br>Mean (range):<br>Prenatal<br>37.74 (7.10 – 62.83) μg/m^3<br>Postnatal	Prenatal<br><br>Postnatal(infancy)	Medical diagnosis of bronchiolitis, bronchitis, or pneumonia, parental report at 2 years of age	Prenatal:<br>0.96, 0.82 – 1.12<br><br>Postnatal:<br>1.04, 0.89 – 1.22	

				37.01 (8.70 – 62.28) μg/m^3				
**Kennedy 2018[6]**	Metropolitan Atlanta, Georgia, U.S.; Cfa	n=22,441, **29–24 months**	Estimated using air pollutants from traffic in the vicinity of a roadway	Log-transformed: Median (range): $PM_{2.5}$ – 1.41 (0.06 – 13.76) μg/m^3 NOx – 0.06 (0.01 – 0.59) ppm CO – 0.59 (0.10 – 5.13) ppm	Postnatal (infancy)	Pneumonia and bronchiolitis (time to first diagnosis), register-based data	<u>Pneumonia</u>: $PM_{2.5}$ –1.04, 0.89 – 1.23 NOx – 1.04, 0.90 – 1.21 CO – (1.03, 0.87 – 1.21) <u>Bronchiolitis:</u> $PM_{2.5}$ – **1.23, 1.15 – 1.32** NOx – **1.19, 1.12 – 1.27** CO – **1.16, 1.08, 1.25**	Data is from subjects with health insurance, only a few were insured
**Soh 2018[7]**	Singapore; Af	n = 953, **0-2** years	Nationwide average, not individual level exposure	$PM_{2.5}$, quartiles Median (IQR) – 17.59 (16.84 – 19.07) μg/m^3	Prenatal	Doctor-diagnosed bronchitis and pneumonia ever, **parental report**	<u>Bronchitis</u> **Q_2** – 0.76, 0.51–1.14 **Q_3** – 1.13 0.78–1.63 **Q_4** – 1.28, 0.88–1.84 <u>Pneumonia</u> **Q_2** – 0.98, 0.61–1.56 **Q_3** – (0.90, 0.56–1.43) **Q_4** – (0.93, 0.50–1.74)	
**Madsen 2017[8]**	Norway; Cfb, Cfc, Dfb, Dfc (main type), Dsb, Dsc, ET	n= 17, 533, **0-18** months	Individual-level exposure	NO_2, per 10 μg/m^3 Mean (SD) -13.6(6.9) μg/m^3	Prenatal	LRTIs, parental report at 6- and 18-months age	<u>0-6months</u> 0.99, 0.84 – 1.17 <u>6-18 months</u> 1.05, 0.94 – 1.16	
**Aguilera, 2013[9]**	Asturias, Gipuzkoa, Sabadell, and Valencia, Spain; Cfb, Cfa, BSk	n = 2199, **12–18** months	Individual-level exposure	NO2, 10-μg/m^3 increase	Prenatal<br><br>Postnatal(infancy)	Doctor-diagnosed LRTIs, parental report	<u>Prenatal</u>: 1.05, 0.98 – 1.12 <u>Postnatal</u>: 1.03, 0.95 – 1.11	No adjustments were made for the other pollutants due to high collinearity.
**Jedrychowski 2013[10]**	Krakow, Poland; Dfb	n=214, **0-7** years	Personal monitoring of two-day measurements, during 2nd trimester.	$PM_{2.5}$ (ln transformed) Mean (SD) - 42.37(27.55) μg/m^3	Prenatal	Recurrent episodes of bronchitis and pneumonia, parental report	**2.05, 1.05–3.99**	

**Esplugues 2011 [11]**	Valencia, Spain; BSk	n= 352, **12 months**	Individual level exposure - **prenatal** (LUR) and **postnatal** (passive sampler at home address)	NO_2, 10 µg/m^3 increase<br>Median (IQR):<br>Prenatal<br>39.4 (31.6 – 48.6) µg/m^3<br>Postnatal<br>26.1 (18.4 - 37.1) µg/m^3	Prenatal<br><br>Postnatal (infancy)	any episode of LRTI during the child's first year of life diagnosed by a doctor (bronchitis, bronchiolitis, or pneumonia), parental report	Prenatal:<br>LRTIs – 1.18, 0.92 - 1.53<br>Bronchiolitis – 1.23, 0.93 – 1.62<br>bronchitis – 1.39, 0.94 – 2.05<br>Postnatal:<br>LRTIs – 0.94, 0.78 - 1.15<br>Bronchiolitis – 0.96, 0.78 – 1.19<br>bronchitis – 1.00, 0.76 – 1.32)	But postnatal NO2 associated with persistent cough (more than 3 weeks) as of parents' report
**Brauer 2007[12]**	Several communities in the Netherlands; Cfb	4th year (n= 2807) ever had outcome (n=2543),<br><br>**birth - 4** years	Individual-level exposure	IQR increase:<br>Median (range) -<br>$PM_{2.5}$<br>17.3(13.5 - 25.2) µg/m^3<br>NO_2<br>26.0(12.6 - 58.4) µg/m^3	Postnatal (lifetime)	Doctor-diagnosed bronchitis, parental report: at 4th year and ever	**at 4th year:**<br>$PM_{2.5}$ – 0.86, 0.66–1.11<br>NO_2 – 0.89, 0.71–1.12<br>**Ever:**<br>$PM_{2.5}$ – 0.96, 0.81–1.13<br>NO_2 – 0.94, 0.82–1.08	
**Brauer 2002[13]**	Northern, western, and central parts of the Netherlands; Cfb	n=2986, **birth - 2** years	Individual-level exposure	IQR increase:<br>Median (range) -<br>$PM_{2.5}$<br>17.3(13.5 - 25.2) µg/m^3<br>NO_2<br>26.0(12.6 - 58.4) µg/m^3	Postnatal (lifetime)	Doctor-diagnosed bronchitis, parental report: at 2nd year	$PM_{2.5}$ – 1.04, 0.85–1.26<br>NO_2 – 0.99, 0.84–1.17	

[a] Climatic zone classification was based on Köppen-Geiger classification-based open data from the Climate Data website (**https://en.climate-data.org/**); The climate zone codes in the table mean the following: Cfb - Temperate oceanic climate, Cfc - Subpolar oceanic climate, Dfb - Warm-summer humid continental climate, Dfc – Subarctic climate, Dsb - Warm, dry-summer continental climate, Dsc - Dry-summer subarctic climate, ET - Tundra, Cfa - Humid subtropical climate, BSk - Cold semi-arid (steppe) climate, BSh - Hot semi-arid (steppe) climate, , Dfa - Hot-summer humid continental climate, Af - Tropical rainforest climate, Csa - Hot-summer Mediterranean climate, Csb - Warm-summer Mediterranean climate

Table S2. ICD Codes and the corresponding Diagnoses

Diagnosis	ICD-8	ICD-9
**Acute bronchitis**		466.0A, 466.1A, 466.99
**Viral pneumonia**		480.1A, 480.8X, 480.9X
**Pneumococcal pneumonia**		481.0A
**Other bacterial pneumonia**	482.98	
**Pneumonia due to another specified organism**		483.0A, 483.0X
**Unspecified pneumonia**	486.09	485.09, 485.9X, 486.11

ICD – International Classification of Diseases

Table S3. Correlation matrix between the two air pollutant exposure periods, pregnancy and the first year of life

Exposure period		First year of life						Pregnancy					
		$PM_{2.5}$	PM_{10}	CO	SO_2	NO_2	O_3	$PM_{2.5}$	PM_{10}	CO	SO_2	NO_2	O_3
First year of life	$PM_{2.5}$	1.0000											
	PM_{10}	0.9966	1.0000										
	CO	0.8767	0.8941	1.0000									
	SO_2	0.9297	0.9496	0.8662	1.0000								
	NO_2	0.9182	0.9357	0.9843	0.9062	1.0000							
	O_3	-0.7440	-0.7290	-0.6535	-0.7265	-0.6752	1.0000						
Pregnancy	$PM_{2.5}$	0.6023	0.6184	0.6364	0.6319	0.6690	-0.3421	1.0000					
	PM_{10}	0.6074	0.6277	0.6484	0.6496	0.6820	-0.3441	0.9969	1.0000				
	CO	0.5382	0.5623	0.6714	0.5717	0.6776	-0.3435	0.8978	0.9067	1.0000			
	SO_2	0.6600	0.6874	0.6664	0.7560	0.7036	-0.4408	0.9028	0.9251	0.8485	1.0000		
	NO_2	0.5783	0.6060	0.6981	0.6181	0.7161	-0.3246	0.9202	0.9353	0.9725	0.8960	1.0000	
	O_3	-0.3342	-0.3271	-0.3176	-0.3284	-0.3117	0.2392	-0.2848	-0.2729	-0.2656	-0.3775	-0.3148	1.0000

Table S4. Average concentrations (µg/m3) of exposures by quartiles during pregnancy and first year period [a]

Pollutants	<Q_1	Q_1-Q_2	Q_2-Q_3	>Q_3
**During entire pregnancy**				
**$PM_{2.5}$**	14.27 (2.84)	18.19 (0.72)	20.82 (0.70)	25.19 (2.98)
**PM_{10}**	15.39 (3.18)	19.74 (0.77)	22.59 (0.76)	27.69 (3.69)
**NO_2**	5.53 (1.69)	8.12 (0.44)	9.57 (0.37)	11.66 (1.28)
**CO**	282.3 (37.93)	345.5 (10.69)	385.4 (11.26)	441.9 (29.79)
**SO_2**	5.53 (1.73)	8.95 (0.91)	11.75 (0.71)	18.14 (5.53)
**O_3**	42.43 (2.42)	48.35 (1.37)	52.67 (1.23)	57.64 (2.31)
**First year of life**				
**$PM_{2.5}$**	14.72 (2.44)	18.01 (0.55)	20.22 (0.61)	23.90 (2.71)
**PM_{10}**	15.93 (2.74)	19.60 (0.57)	22.01 (0.67)	26.26 (3.33)
**NO_2**	5.85 (1.52)	8.08 (0.45)	9.49 (0.33)	11.20 (1.19)
**CO**	291.4 (34.59)	346.9 (13.49)	383.7 (8.35)	427.4 (26.68)
**SO_2**	5.52 (1.48)	8.54 (0.55)	11.21 (0.76)	16.44 (4.82)
**O_3**	47.67 (1.32)	49.94 (0.54)	51.92 (0.62)	54.68 (1.88)

[a] Mean (Standard deviation); Q_1, Q_2 and Q_3 represent quartiles 1, 2 and 3, respectively (the corresponding values are presented in Table 2).

Table S5: Incidence rates of lower respiratory tract infections during the first two-years of life by sex, the Espoo Cohort Study, 1991 – 2011.[a]

Infections	Sex	n	IR (95% CI)	IRR (95% CI)
**0–1 year**				
LRTIs	Girl	8	0.64 (0.27, 1.25)	1.00
	Boy	18	1.37 (0.81, 2.17)	2.16 (0.89, 5.73)
	**Total**	**26**	**1.01 (0.66, 1.48)**	-
Acute bronchitis	Girl	3	0.24 (0.05, 0.70)	1.00
	Boy	8	0.61(0.26, 1.20)	2.56 (0.61, 14.96)
	Total	11	0.43 (0.21, 0.77)	-
Pneumonia	Girl	5	0.40 (0.13, 0.93)	1.00
	Boy	10	0.76 (0.37, 1.40)	1.29 (0.78, 2.16)
	Total	15	0.58 (0.32, 0.96)	-
**1–2 years**				
LRTIs	Girl	29	2.31 (1.55, 3.31)	1.00
	Boy	34	2.59 (1.79, 3.62)	1.12 (0.66, 1.91)
	**Total**	**63**	**2.45 (1.89, 3.14)**	-
Acute bronchitis	Girl	5	0.40 (0.13, 0.93)	1.00
	Boy	5	0.38 (0.12, 0.89)	0.96 (0.22, 4.17)
	Total	10	0.39 (0.19, 0.72)	-
Pneumonia	Girl	24	1.91 (1.22, 2.84)	1.00
	Boy	29	2.21 (1.48, 3.18)	1.16 (0.65, 2.08)
	Total	53	2.06 (1.55, 2.70)	-
**0–2 years**				
LRTIs	Girl	37	1.47 (1.04, 2.6)	1.00
	Boy	52	1.98 (1.48, 2.60)	1.35 (0.87, 2.11)
	**Total**	**89**	**1.73 (1.39, 2.13)**	-
Acute bronchitis	Girl	8	0.32 (0.14, 0.63)	1.00
	Boy	13	0.50 (0.26, 0.85)	1.56 (0.60, 4.34)
	Total	21	0.41 (0.25, 0.63)	-
Pneumonia [b]	Girl	29	1.15 (0.77, 1.66)	1.00
	Boy	39	1.49 (1.06, 2.03)	1.29 (0.78, 2.16)
	Total	68	1.32 (1.03, 1.68)	-

n – episodes of infections; IR – Incidence rate per 100 person-years; CI – Confidence interval; IRR – Incidence rate ratio. LRTIs – Lower Respiratory Tract Infections.

[a] Incidence rate ratios (IRRs) were calculated based on IR among men divided by IR among women.

[b] Pneumonias included i) viral pneumonia (n = 5), ii) bacterial pneumonia (n = 1), iii) pneumonia by other specified organism (n = 1), and unspecified pneumonia (n = 46).

Table S6. Association between exposure to air pollution during the entire pregnancy and lower respiratory tract infections during the first two years of life

**Exposure**	**Effect estimates compared to the first quartile, IRR (95% CI)**			**Per 10 μg/m^3 of air pollutant exposure**
	Quartile 2	Quartile 3	Quartile 4	IRR (95% CI)
**$PM_{2.5}$ [a]**	1.48 (0.79, 2.76)	0.87 (0.44, 1.72)	0.87 (0.43, 1.73)	0.93 (0.52, 1.66)
**$PM_{2.5}$ [b]**	1.58 (0.83, 2.98)	0.87 (0.43, 1.76)	0.61 (0.28, 1.31)	0.53 (0.28, 0.99)
**+SO_2**	1.51 (0.78, 2.93)	0.77 (0.32, 1.85)	0.51 (0.17, 1.53)	0.54 (0.17, 1.71)
**+O_3**	1.64 (0.87, 3.10)	0.87 (0.43, 1.76)	0.58 (0.27, 1.25)	0.50 (0.27, 0.93)
**+SO_2 +O_3**	1.67 (0.85, 3.28)	0.91 (0.37, 2.20)	0.62 (0.20, 1.90)	0.68 (0.21, 2.22)
**NO_2 [a]**	1.28 (0.70, 2.35)	0.45 (0.21, 0.97)	0.68 (0.33, 1.37)	0.76 (0.23, 2.52)
**NO_2 [b]**	1.42 (0.76, 2.64)	0.45 (0.20, 0.98)	0.46 (0.21, 1.01)	0.31 (0.08, 1.13)
**+ PM_{10}**	1.44 (0.72, 2.90)	0.46 (0.16, 1.33)	0.48 (0.13, 1.79)	1.33 (0.09, 20.34)
**+ O_3**	1.32 (0.71, 2.46)	0.43 (0.20, 0.93)	0.42 (0.20, 0.91)	0.27 (0.07, 0.96)
**+ PM_{10}+ O_3**	1.33 (0.66, 2.67)	0.43 (0.15, 1.23)	0.42 (0.11, 1.56)	0.97 (0.06, 15.77)
**CO [a]**	0.89 (0.47, 1.69)	0.69 (0.34, 1.38)	0.75 (0.37, 1.52)	1.00 (0.95, 1.04)
**CO [b]**	0.92 (0.48, 1.77)	0.59 (0.28, 1.23)	0.49 (0.23, 1.05)	0.96 (0.91, 1.01)
**+ PM_{10}**	1.06 (0.52, 2.18)	0.82 (0.30, 2.27)	0.78 (0.22, 2.77)	1.01 (0.92, 1.10)
**+ O_3**	0.90 (0.47, 1.71)	0.56 (0.27, 1.17)	0.46 (0.21, 0.99)	0.96 (0.91, 1.00)
**+ SO_2**	0.83 (0.40, 1.74)	0.47 (0.17, 1.32)	0.36 (0.10, 1.28)	0.97 (0.91, 1.05)
**+ SO_2 + O_3**	0.91 (0.43, 1.91)	0.58 (0.20, 1.68)	0.48 (0.13, 1.82)	0.99 (0.92, 1.07)
**+ PM_{10}+ O_3**	1.05 (0.51, 2.16)	0.81 (0.29, 2.26)	0.77 (0.21, 2.81)	1.01 (0.92, 1.11)
**PM_{10} [a]**	1.18 (0.64, 2.18)	0.79 (0.41, 1.51)	0.71 (0.36, 1.40)	0.93 (0.55, 1.56)
**PM_{10} [b]**	1.29 (0.69, 2.41)	0.79 (0.40, 1.55)	0.48 (0.22, 1.03)	0.55 (0.31, 0.98)
**+ CO**	1.32 (0.66, 2.63)	0.83 (0.32, 2.16)	0.51 (0.15, 1.74)	0.41 (0.12, 1.34)
**+ NO_2**	1.47 (0.74, 2.93)	1.05 (0.41, 2.67)	0.73 (0.21, 2.59)	0.40 (0.10, 1.51)
**+ CO + O_3**	1.39 (0.69, 2.77)	0.83 (0.32, 2.15)	0.50 (0.14, 1.71)	0.41 (0.12, 1.34)
**+ NO_2 + O_3**	1.63 (0.81, 3.28)	1.15 (0.45, 2.89)	0.83 (0.23, 2.94)	0.46 (0.12, 1.75)
**SO_2 [a]**	2.43 (1.21, 4.88)	1.21 (0.55, 2.69)	0.92 (0.40, 2.09)	0.80 (0.44, 1.45)
**SO_2 [b]**	2.70 (1.32, 5.52)	1.09 (0.47, 2.53)	0.72 (0.30, 1.74)	0.50 (0.24, 1.01)
**+ CO**	3.08 (1.42, 6.71)	1.52 (0.49, 4.71)	1.16 (0.28, 4.73)	0.50 (0.24, 1.01)
**+ NO_2**	3.66 (1.66, 8.07)	2.21 (0.69, 7.04)	2.13 (0.47, 9.68)	0.49 (0.12, 1.92)
**+ O_3**	2.39 (1.16, 4.96)	0.98 (0.42, 2.29)	0.60 (0.24, 1.49)	0.42 (0.20, 0.87)
**+ CO + O_3**	2.60 (1.16, 5.84)	1.18 (0.36, 3.86)	0.79 (0.18, 3.58)	0.32 (0.08, 1.25)
**+NO_2 + O_3**	3.19 (1.41, 7.22)	1.85 (0.57, 6.07)	1.62 (0.33, 7.85)	0.34 (0.08, 1.54)
**O_3 [a]**	0.79 (0.45, 1.39)	0.46 (0.23, 0.90)	1.11 (0.63, 1.95)	1.00 (0.69, 1.44)
**O_3 [b]**	0.69 (0.38, 1.25)	0.46 (0.23, 0.90)	0.75 (0.40, 1.40)	0.75 (0.50, 1.11)
**+ SO_2**	0.68 (0.38, 1.23)	0.43 (0.21, 0.85)	0.65 (0.34, 1.24)	0.64 (0.42, 0.97)
**+ NO_2**	0.69 (0.38, 1.24)	0.43 (0.22, 0.85)	0.64 (0.34, 1.21)	0.68 (0.45, 1.02)
**+ CO**	0.71 (0.39, 1.28)	0.45 (0.23, 0.89)	0.69 (0.37, 1.30)	0.70 (0.47, 1.05)
**+ $PM_{2.5}$**	0.68 (0.38, 1.23)	0.44 (0.22, 0.87)	0.68 (0.36, 1.29)	0.69 (0.46, 1.03)
**+ SO_2 + CO**	0.71 (0.39, 1.29)	0.45 (0.23, 0.91)	0.70 (0.36, 1.36)	0.64 (0.42, 0.97)
**+ PM_{10} + NO_2**	0.69 (0.38, 1.24)	0.43 (0.22, 0.85)	0.64 (0.34, 1.22)	0.70 (0.46, 1.05)
**+ $PM_{2.5}$ + SO_2**	0.68 (0.38, 1.23)	0.44 (0.22, 0.87)	0.68 (0.35, 1.31)	0.65 (0.42, 0.99)
**+ NO_2 + SO_2**	0.70 (0.39, 1.27)	0.45 (0.23, 0.90)	0.69 (0.36, 1.33)	0.63 (0.42, 0.97)

a – single pollutant models, adjusted for prenatal exposure only

b – adjusted for prenatal exposure of the respective pollutant and for sex, breastfeeding duration, maternal smoking during pregnancy, family socioeconomic status, child atopy, and parental atopy.

Table S7. Association between exposure to air pollution during the first year of life and incidence rate of lower respiratory tract infections during the second (1-2) year of life

**Exposure**	**Effect estimates, IRR (95%CI), relative to the first quartile**			**Per 10 μg/ increase**
	Quartile 2	Quartile 3	Quartile 4	IRR (95% CI)
**$PM_{2.5}$ [a]**	0.82(0.34, 1.98)	1.50 (0.66, 3.45)	**2.49 (1.16, 5.34)**	1.30 (0.59, 2.86)
**$PM_{2.5}$ [b]**	0.94 (0.38, 2.32)	1.46 (0.60, 3.58)	**2.95 (1.32, 6.56)**	1.95 (0.86, 4.44)
**+SO_2**	1.59 (0.50, 5.05)	1.96 (0.44, 8.72)	2.09 (0.40, 10.89)	1.65 (0.26, 10.40)
**+O_3**	0.94 (0.33, 2.66)	1.22 (0.36, 4.11)	2.22 (0.63, 7.88)	0.75 (0.25, 2.24)
**+SO_2 +O_3**	1.59 (0.44, 5.79)	1.68 (0.31, 9.10)	1.77 (0.27, 11.44)	0.97 (0.17, 5.48)
**PM_{10} [a]**	0.68 (0.28, 1.68)	1.62 (0.73, 3.58)	**2.25 (1.05, 4.83)**	1.24 (0.61, 2.52)
**PM_{10} [b]**	0.76 (0.30, 1.91)	1.56 (0.67, 3.64)	**2.62 (1.18, 5.83)**	1.80 (0.86, 3.77)
**+ CO**	1.26 (0.44, 3.64)	3.33 (0.97, 11.42)	5.76 (1.46, 22.69)	2.43 (0.66, 8.89)
**+ NO_2**	1.46 (0.48, 4.38)	4.33 (1.14, 16.42)	5.86 (1.22, 28.22)	3.04 (0.57, 16.19)
**+ CO + O_3**	1.09 (0.32, 3.74)	2.38 (0.51, 11.15)	3.41 (0.59, 19.70)	1.16 (0.28, 4.77)
**+ NO_2 + O_3**	1.23 (0.35, 4.40)	3.08 (0.61, 15.56)	3.35 (0.49, 22.83)	1.48 (0.25, 8.74)
**NO_2 [a]**	0.72 (0.32, 1.66)	1.67 (0.73, 3.81)	**2.41 (1.12, 5.19)**	1.63 (0.33, 8.10)
**NO_2 [b]**	0.79 (0.34, 1.86)	1.54 (0.63, 3.77)	**3.03 (1.37, 6.73)**	3.18 (0.57, 17.71)
**+ PM_{10}**	0.42 (0.13, 1.31)	0.42 (0.09, 1.84)	0.68 (0.13, 3.62)	0.56 (0.01, 33.06)
**+ O_3**	0.74 (0.30, 1.82)	1.03 (0.36, 2.97)	1.71 (0.54, 5.36)	0.43 (0.05, 3.91)
**+ PM10+ O_3**	0.47 (0.14, 1.52)	0.45 (0.10, 2.10)	0.75 (0.13, 4.45)	0.26 (0.003, 18.53)
**CO [a]**	0.95 (0.44, 2.06)	1.22 (0.53, 2.80)	1.65 (0.73, 3.73)	1.00 (0.95, 1.07)
**CO [b]**	0.87 (0.37, 2.02)	1.52 (0.64, 3.61)	**2.25 (0.97, 5.23)**	1.04 (0.97, 1.11)
**+ PM_{10}**	0.47 (0.16, 1.40)	0.48 (0.13, 1.76)	0.47 (0.11, 1.98)	0.99 (0.88, 1.11)
**+ O_3**	0.73 (0.30, 1.75)	0.94 (0.35, 2.52)	1.08 (0.38, 3.08)	0.97 (0.89, 1.05)
**+ SO_2**	0.48 (0.14, 1.61)	0.31 (0.07, 1.30)	0.17 (0.03, 0.85)	0.99 (0.90, 1.10)
**+ SO_2 + O_3**	0.53 (0.16, 1.78)	0.34 (0.08, 1.51)	0.18 (0.04, 0.95)	0.97 (0.87, 1.08)
**+ PM_{10}+ O_3**	0.51 (0.17, 1.57)	0.52 (0.13, 1.98)	0.51 (0.11, 2.28)	0.97 (0.86, 1.10)
**SO_2 [a]**	0.44 (0.18, 1.11)	1.09 (0.46, 2.58)	**2.31 (1.05, 5.07)**	1.45 (0.68, 3.09)
**SO_2 [b]**	0.47 (0.19, 1.22)	1.05 (0.42, 2.64)	**2.66 (1.17, 6.05)**	2.12 (0.92, 4.92)
**+ CO**	0.91 (0.27, 3.05)	3.18 (0.79, 12.85)	13.85 (2.89, 66.35)	3.02 (1.03, 8.88)
**+ NO_2**	0.96 (0.28, 3.28)	5.17 (1.30, 20.59)	23.21 (4.21, 128.02)	3.42 (1.04, 11.29)
**+ O_3**	0.47 (0.18, 1.24)	0.93 (0.31, 2.78)	2.27 (0.71, 7.26)	0.89 (0.28, 2.84)
**+ CO + O_3**	0.78 (0.22, 2.72)	2.49 (0.52, 11.88)	10.73 (1.76, 65.39)	1.49 (0.40, 5.57)
**+NO_2 + O_3**	0.85 (0.24, 2.93)	4.05 (0.90, 18.12)	16.65 (2.56, 108.26)	1.69 (0.41, 7.01)
**O_3 [a]**	0.79 (0.43, 1.46)	0.41 (0.19, 0.91)	0.49 (0.23, 1.02)	0.42 (0.16, 1.09)
**O_3 [b]**	0.78 (0.41, 1.49)	0.47 (0.20, 1.09)	0.54 (0.25, 1.16)	0.48 (0.17, 1.32)
**+ SO_2**	1.13 (0.53, 2.39)	0.72 (0.25, 2.07)	0.73 (0.23, 2.31)	0.18 (0.04, 0.85)
**+ NO_2**	0.80 (0.36, 1.78)	0.44 (0.16, 1.24)	0.42 (0.14, 1.24)	0.18 (0.05, 0.69)
**+ CO**	0.69 (0.33, 1.45)	0.40 (0.16, 1.05)	0.39 (0.14, 1.06)	0.18(0.05, 0.69)
**+ $PM_{2.5}$**	1.06 (0.50, 2.25)	0.71 (0.23, 2.14)	0.72 (0.21, 2.52)	0.20 (0.05, 0.82)
**+ SO_2 + CO**	1.08 (0.50, 2.30)	0.80 (0.28, 2.24)	0.79 (0.25, 2.47)	0.18 (0.04, 0.77)
**+ PM_{10} + NO_2**	0.87 (0.38, 1.97)	0.61 (0.20, 1.83)	0.59 (0.16, 2.10)	0.19 (0.05, 0.81)
**+ $PM_{2.5}$ + SO_2**	1.21 (0.55, 2.65)	0.77 (0.25, 2.40)	0.78 (0.22, 2.84)	0.17 (0.04, 0.79)
**+ NO_2 + SO_2**	0.96 (0.44, 2.10)	0.72 (0.25, 2.10)	0.71 (0.22, 2.28)	0.18 (0.04, 0.80)

IRR – Incidence rate ratio; CI – confidence interval; $PM_{2.5}$ – particulate matter with a diameter of 2.5 μm; NO_2 – nitrogen dioxide; CO – carbon monoxide; PM_{10} – particulate matter with a diameter of 10 μm; SO_2 – sulfur dioxide; O_3 – ozone.

a – single pollutant models, adjusted only for prenatal exposure of respective pollutant

b – adjusted for prenatal exposure of respective pollutants and sex, breastfeeding duration, maternal smoking during pregnancy, family socioeconomic status, child atopy and parental atopy.

Table S8. Relation between exposure to air pollution during the first year of life and lower respiratory tract infections during the first two years of life, in term-born babies (n = 2301)

Exposure	IRR (95% CI) of LRTI relative to the first quartile, in term born babies		
	Quartile 2	Quartile 3	Quartile 4
**$PM_{2.5}$ [a]**	1.17 (0.55, 2.50)	1.64 (0.74, 3.60)	**2.67 (1.31, 5.44)**
**$PM_{2.5}$ [b]**	1.33 (0.61, 2.89)	1.85 (0.82, 4.16)	**3.10 (1.47, 6.52)**
**$+SO_2$**	1.93 (0.69, 5.38)	2.06 (0.56, 7.62)	1.53 (0.35, 6.69)
**$+O_3$**	1.46 (0.60, 3.52)	1.76 (0.60, 5.20)	2.71 (0.87, 8.44)
**$+SO_2$ $+O_3$**	2.12 (0.68, 6.60)	1.97 (0.45, 8.68)	1.47 (0.28, 7.81)
**NO_2 [a]**	0.71 (0.33, 1.51)	1.58 (0.74, 3.38)	**2.38 (1.17, 4.85)**
**NO_2 [b]**	0.76 (0.35, 1.64)	1.73 (0.79, 3.77)	**2.78 (1.33, 5.84)**
**$+ PM_{10}$**	0.37 (0.14, 0.96)	0.65 (0.20, 2.16)	0.98 (0.24, 4.04)
**$+ O_3$**	0.76 (0.33, 1.75)	1.43 (0.56, 3.62)	2.15 (0.77, 6.04)
**$+ PM_{10}+ O_3$**	0.40 (0.15, 1.10)	0.70 (0.21, 2.40)	1.11 (0.25, 4.95)
**CO [a]**	0.78 (0.36, 1.66)	1.55 (0.73, 3.29)	1.97 (0.92, 4.20)
**CO [b]**	0.88 (0.41, 1.91)	1.76 (0.81, 3.82)	**2.27 (1.02, 5.01)**
**$+ PM_{10}$**	0.46 (0.18, 1.18)	0.71 (0.24, 2.04)	0.67 (0.19, 2.32)
**$+ O_3$**	0.80 (0.36, 1.79)	1.35 (0.57, 3.22)	1.45 (0.56, 3.77)
**$+ SO_2$**	0.49 (0.17, 1.39)	0.54 (0.16, 1.83)	0.28 (0.07, 1.16)
**$+ SO_2 + O_3$**	0.55 (0.19, 1.60)	0.64 (0.18, 2.26)	0.32 (0.08, 1.37)
**$+ PM_{10}+ O_3$**	0.50 (0.19, 1.31)	0.76 (0.25, 2.31)	0.74 (0.20, 2.68)
**PM_{10} [a]**	1.23 (0.57, 2.66)	1.80 (0.83, 3.92)	**2.80 (1.35, 5.81)**
**PM_{10} [b]**	1.38 (0.63, 3.02)	1.96 (0.88, 4.35)	**3.20 (1.50, 6.84)**
**+ CO**	2.19 (0.87, 5.52)	**3.32 (1.10, 10.01)**	**4.95 (1.49, 16.46)**
**$+ NO_2$**	**2.69 (1.03, 6.99)**	**4.01 (1.21, 13.30)**	**4.63 (1.18, 18.25)**
**$+ CO + O_3$**	1.63 (0.59, 4.53)	1.96 (0.53, 7.27)	2.62 (0.60, 11.44)
**$+ NO_2 + O_3$**	2.78 (0.90, 8.56)	3.82 (0.88, 16.70)	3.89 (0.88, 21.62)
**SO_2 [a]**	0.70 (0.32, 1.53)	1.00 (0.43, 2.33)	**2.46 (1.16, 5.22)**
**SO_2 [b]**	0.74 (0.33, 1.63)	1.08 (0.45, 2.57)	**2.97 (1.36, 6.45)**
**+ CO**	1.29 (0.46, 3.67)	2.19 (0.60, 8.06)	**8.64 (2.13, 34.96)**
**$+ NO_2$**	1.56 (0.54, 4.49)	3.08 (0.77, 12.37)	**12.36 (2.53, 60.46)**
**$+ O_3$**	0.75 (0.33, 1.71)	1.05 (0.38, 2.92)	2.90 (0.98, 8.58)
**$+ CO + O_3$**	1.12 (0.37, 3.42)	1.83 (0.41, 8.24)	**7.61 (1.41, 41.09)**
**$+NO_2 + O_3$**	1.39 (0.45, 4.24)	2.63 (0.57, 12.23)	**10.36 (1.72, 62.47)**
**O_3 [a]**	0.86 (0.48, 1.54)	0.45 (0.22, 0.95)	0.59 (0.30, 1.15)
**O_3 [b]**	0.89 (0.48, 1.62)	0.51 (0.24, 1.08)	0.59 (0.29, 1.18)
**$+ SO_2$**	1.29 (0.64, 2.58)	0.80 (0.32, 2.04)	0.88 (0.32, 2.44)
**$+ NO_2$**	1.00 (0.49, 2.02)	0.58 (0.24, 1.41)	0.59 (0.23, 1.52)
**+ CO**	0.88 (0.45, 1.71)	0.51 (0.22, 1.18)	0.54 (0.23, 1.31)
**$+ PM_{2.5}$**	1.09 (0.55, 2.16)	0.69 (0.26, 1.77)	0.86 (0.30, 2.48)
**$+ SO_2 + CO$**	1.26 (0.62, 2.55)	0.85 (0.34, 2.13)	0.95 (0.35, 2.63)
**$+ PM_{10} + NO_2$**	1.01 (0.49, 2.09)	0.68 (0.26, 1.73)	0.92 (0.32, 2.64)
**$+ PM_{2.5} + SO_2$**	1.26 (0.61, 2.60)	0.76 (0.28, 2.03)	0.93 (0.31, 2.77)
**$+ NO_2 + SO_2$**	1.15 (0.56, 2.34)	0.82 (0.32, 2.09)	0.89 (0.32, 2.48)

a – single pollutant models, adjusted only for prenatal exposure

b – adjusted for prenatal exposure of respective pollutant and sex, breastfeeding duration, maternal smoking during pregnancy, family socioeconomic status, child atopy and parental atopy.

Table S9. Incidence rate ratio for the relation between exposure to air pollution during the first year of life and lower respiratory tract infections during the first two years of life,

Exposure	Effect estimates relative to the first quartile, girls			Effect estimates relative to the first quartile, boys		
	Quartile 2	Quartile 3	Quartile 4	Quartile 2	Quartile 3	Quartile 4
**$PM_{2.5}$ [a]**	0.91 (0.31, 2.71)	1.10 (0.35, 3.48)	**2.86 (1.10, 7.43)**	1.54 (0.57, 4.15)	2.42 (0.93, 6.31)	2.26 (0.86, 5.89)
**$PM_{2.5}$ [b]**	1.05 (0.35, 3.12)	1.16 (0.35, 3.90)	**3.71 (1.38, 9.98)**	1.74 (0.62, 4.90)	2.13 (0.75, 6.09)	**2.76 (1.01, 7.57)**
**NO_2 [a]**	0.26 (0.07, 0.96)	1.12 (0.39, 3.20)	2.40 (0.98, 5.85)	1.63 (0.63, 4.24)	**2.88 (1.07, 7.80)**	2.46 (0.91, 6.70)
**NO_2 [b]**	0.28 (0.08, 1.01)	1.66 (0.57, 4.83)	**2.78 (1.09, 7.09)**	1.82 (0.67, 4.97)	2.32 (0.78, 6.82)	**3.43 (1.20, 9.81)**
**CO [a]**	0.26 (0.07, 0.95)	1.10 (0.42, 2.85)	1.07 (0.38, 3.01)	2.12 (0.86, 5.25)	2.05 (0.73, 5.76)	**2.83 (1.06, 7.57)**
**CO [b]**	0.30 (0.08, 1.09)	1.52 (0.58, 3.98)	1.11 (0.36, 3.42)	2.01 (0.75, 5.42)	2.17 (0.71, 6.65)	**4.58 (1.64, 12.76)**
**PM_{10} [a]**	1.11 (0.37, 3.34)	1.44 (0.47, 4.38)	**3.21 (1.20, 8.56)**	1.12 (0.42, 2.97)	2.10 (0.84, 5.25)	1.91 (0.75, 4.84)
**PM_{10} [b]**	1.24 (0.41, 3.75)	1.44 (0.46, 4.56)	**3.88 (1.41, 10.69)**	1.22 (0.44, 3.36)	1.74 (0.64, 4.75)	2.33 (0.88, 6.17)
**SO_2 [a]**	0.78 (0.25, 2.38)	1.03 (0.30, 3.53)	**2.85 (0.97, 8.33)**	0.74 (0.28, 1.97)	1.27 (0.48, 3.38)	2.22 (0.88, 5.61)
**SO_2 [b]**	0.84 (0.28, 2.54)	1.45 (0.42, 5.00)	**3.64 (1.21, 10.98)**	0.76 (0.28, 2.08)	1.04 (0.36, 3.00)	2.48 (0.94, 6.55)
**O_3 [a]**	0.78 (0.36, 1.72)	0.21 (0.06, 0.75)	0.44 (0.17, 1.14)	0.87 (0.43, 1.77)	0.79 (0.36, 1.73)	0.55 (0.23, 1.27)
**O_3 [b]**	1.04 (0.45, 2.40)	0.28 (0.08, 1.04)	0.52 (0.19, 1.41)	0.66 (0.31, 1.43)	0.76 (0.33, 1.72)	0.55 (0.23, 1.32)

a – single pollutant models, adjusted only for prenatal exposure

b – adjusted for prenatal exposure of the studied pollutant, duration of breastfeeding, maternal smoking during pregnancy, family socioeconomic status, child atopy, and parental atopy.

Appendix 2: Weighted Quantile Sum regression

Weighted Quantile Sum (WQS) regression analysis is a statistical method for estimating the effect of simultaneous exposure to multiple environmental pollutants on health. This analysis is conducted by splitting the data into training and validation data sets. It was conducted in two steps using the R package, "gWQS–generalized weighted quantile sum regression." First, the model estimates the WQS index, a mixture variable, using the training data set (40% in our data) and bootstrap sampling (n =200). The weighted quantile sum index is a weighted sum of each pollutant's quantile (i.e., in this case, quartiles) based on each pollutant's relevance to the outcome (i.e., the incidence of LRTIs) derived from bootstrap samples. In the second step, the model estimates the cumulative effect of the air pollutant mixture on the outcome. A positive relationship was detected between exposure to air pollutants and the incidence of LRTIs, as indicated in Figure 1. Therefore, we assumed a positive association between exposure to air pollutants and the incidence of LRTIs to estimate the effect of the mixture.

The effect of exposure to ambient air pollutant mixture during pregnancy and the first year of life on the incidence rate of LRTIs during the first two years of life was modeled using WQS regression as follows:

$$g(\mu) = \beta_0 + \beta_1 \left(\sum_{i=1}^{C} \omega_i q_i \right) + z'\varphi$$

g Poisson function with log link. μ represents the mean number LRTIs during the first two years of life. β_0 is the intercept. ω_i is the unknown weight for the ith exposure. q_i represents the quartile of exposure (e.g., qi = 0, 1, 2, or 3 for values in the 1st, 2nd, 3rd, or 4th quartile, respectively). C is the number of exposures. $\sum_{i=1}^{C} \omega_i q_i$ represents the weighted index for the set of c pollutants of interest,

where $\sum_{i=1}^{C} \omega_i = 1$ and $0 \leq \omega_i \leq 1$. β_1 is the regression coefficient of the weighted quantile sum. z is a vector of covariates. φ is a vector of regression coefficients for the covariates.

References

1 Lu C, Yang W, Wang F, *et al.* Effects of intrauterine and post-natal exposure to air pollution on children's pneumonia: Key roles in different particulate matters exposure during critical time windows. *J Hazard Mater*. 2023;457:131837.

2 Goshen S, Novack L, Erez O, *et al.* The effect of exposure to particulate matter during pregnancy on lower respiratory tract infection hospitalizations during first year of life. *Env Health*. 2020;19:90.

3 Yang S, Kim H, Kim H, *et al.* Particulate matter at third trimester and respiratory infection in infants, modified by *GSTM1*. *Pediatr Pulmonol*. 2020;55:245–53.

4 Liu W, Huang C, Cai J, *et al.* Prenatal and postnatal exposures to ambient air pollutants associated with allergies and airway diseases in childhood: A retrospective observational study. *Environ Int*. 2020;142:105853.

5 Gutiérrez Oyarce A, Ferrero A, Estarlich M, *et al.* [Exposure to nitrogen dioxide and respiratory health at 2 years in the INMA-Valencia cohort]. *Gac Sanit*. 2018;32:507–12.

6 Kennedy CM, Pennington AF, Darrow LA, *et al.* Associations of mobile source air pollution during the first year of life with childhood pneumonia, bronchiolitis, and otitis media. *Env Epidemiol*. 2018;2:e007.

7 Soh SE, Goh A, Teoh OH, *et al.* Pregnancy Trimester-Specific Exposure to Ambient Air Pollution and Child Respiratory Health Outcomes in the First 2 Years of Life: Effect Modification by Maternal Pre-Pregnancy BMI. *Int J Env Res Public Health*. 2018;15. doi: 10.3390/ijerph15050996

8 Madsen C, Haberg SE, Magnus MC, *et al.* Pregnancy exposure to air pollution and early childhood respiratory health in the Norwegian Mother and Child Cohort Study (MoBa). *BMJ Open*. 2017;7:e015796.

9 Aguilera I, Pedersen M, Garcia-Esteban R, *et al.* Early-life exposure to outdoor air pollution and respiratory health, ear infections, and eczema in infants from the INMA study. *Env Health Perspect*. 2013;121:387–92.

10 Jedrychowski WA, Perera FP, Spengler JD, *et al.* Intrauterine exposure to fine particulate matter as a risk factor for increased susceptibility to acute broncho-pulmonary infections in early childhood. *Int J Hyg Env Health*. 2013;216:395–401.

11 Esplugues A, Ballester F, Estarlich M, *et al.* Outdoor, but not indoor, nitrogen dioxide exposure is associated with persistent cough during the first year of life. *Sci Total Env*. 2011;409:4667–73.

12 Brauer M, Hoek G, Smit HA, *et al.* Air pollution and development of asthma, allergy and infections in a birth cohort. *Eur Respir J*. 2007;29:879–88.

13 Brauer M, Hoek G, Van Vliet P, *et al.* Air Pollution from Traffic and the Development of Respiratory Infections and Asthmatic and Allergic Symptoms in Children. *Am J Respir Crit Care Med*. 2002;166:1092–8.
